# 2-(3,5-Dimethyl-1*H*-pyrazol-1-yl)-2-hy­droxy­imino-*N*′-[1-(pyridin-2-yl)ethyl­idene]acetohydrazide

**DOI:** 10.1107/S1600536812045412

**Published:** 2012-11-17

**Authors:** Maxym O. Plutenko, Rostislav D. Lampeka, Matti Haukka, Ebbe Nordlander

**Affiliations:** a Department of Chemistry, National Taras Shevchenko University, Volodymyrska Street 64, 01601 Kyiv, Ukraine; b Department of Chemistry, University of Jyvaskyla, PO Box 35, FI-40014 Jyvaskyla, Finland; c Inorganic Chemistry, Center for Chemistry and Chemical Engineering, Lund University, Box 124, SE-221 00 Lund, Sweden

## Abstract

In the title compound, C_14_H_16_N_6_O_2_, the dihedral angles formed by the mean plane of the acetohydrazide group [maximum deviation 0.0629 (12) Å] with the pyrazole and pyridine rings are 81.62 (6) and 38.38 (4)° respectively. In the crystal, mol­ecules are connected by N—H⋯O and O—H⋯N hydrogen bonds into supra­molecular chains extending parallel to the *c*-axis direction.

## Related literature

For uses of polynuclear complexes, see: Świątek-Kozłowska *et al.* (2000 ▶); Wörl *et al.* (2005 ▶). For the use of oximes having additional donor functions as versatile ligands, see: Krämer & Fritsky (2000 ▶); Sachse *et al.* (2008 ▶); Kanderal *et al.* (2005 ▶). For related structures, see: Moroz *et al.* (2012 ▶); Mokhir *et al.* (2002 ▶); Sliva *et al.* (1997 ▶). For the synthesis, see: Kozikowski & Adamczyk (1983 ▶).
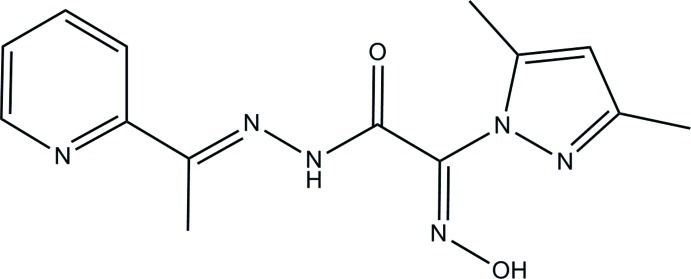


## Experimental

### 

#### Crystal data

C_14_H_16_N_6_O_2_
*M* *_r_* = 300.33
Monoclinic, 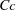

*a* = 24.5792 (6) Å
*b* = 7.5795 (2) Å
*c* = 8.3072 (2) Å
β = 107.335 (1)°
*V* = 1477.32 (6) Å^3^
*Z* = 4
Mo *K*α radiation
μ = 0.10 mm^−1^
*T* = 100 K
0.36 × 0.28 × 0.21 mm

#### Data collection

Bruker Kappa APEXII DUO CCD diffractometer
Absorption correction: multi-scan (*SADABS*; Sheldrick, 2008 ▶) *T*_min_ = 0.966, *T*_max_ = 0.980
8846 measured reflections
4395 independent reflections
4096 reflections with *I* > 2σ(*I*)
*R*_int_ = 0.016

#### Refinement

*R*[*F* ^2^ > 2σ(*F* ^2^)] = 0.032
*wR*(*F* ^2^) = 0.081
*S* = 1.03
4395 reflections
204 parameters
3 restraints
H-atom parameters constrained
Δρ_max_ = 0.36 e Å^−3^
Δρ_min_ = −0.24 e Å^−3^

### 

Data collection: *APEX2* (Bruker, 2010 ▶); cell refinement: *SAINT* (Bruker, 2009 ▶); data reduction: *SAINT*; program(s) used to solve structure: *SHELXS97* (Sheldrick, 2008 ▶); program(s) used to refine structure: *SHELXL97* (Sheldrick, 2008 ▶); molecular graphics: *DIAMOND* (Brandenburg, 2009 ▶); software used to prepare material for publication: *SHELXL97*.

## Supplementary Material

Crystal structure: contains datablock(s) I, global. DOI: 10.1107/S1600536812045412/rz5011sup1.cif

Supplementary material file. DOI: 10.1107/S1600536812045412/rz5011Isup2.mol

Structure factors: contains datablock(s) I. DOI: 10.1107/S1600536812045412/rz5011Isup3.hkl

Supplementary material file. DOI: 10.1107/S1600536812045412/rz5011Isup4.cml

Additional supplementary materials:  crystallographic information; 3D view; checkCIF report

## Figures and Tables

**Table 1:** Hydrogen-bond geometry (Å, °)

*D*—H⋯*A*	*D*—H	H⋯*A*	*D*⋯*A*	*D*—H⋯*A*
O2—H2*O*⋯N6^i^	0.88	1.79	2.6686 (13)	175
N3—H3*N*⋯O1^i^	0.86	2.17	3.0196 (13)	174

Symmetry code: (i) 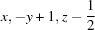.

**Fig. 1.**
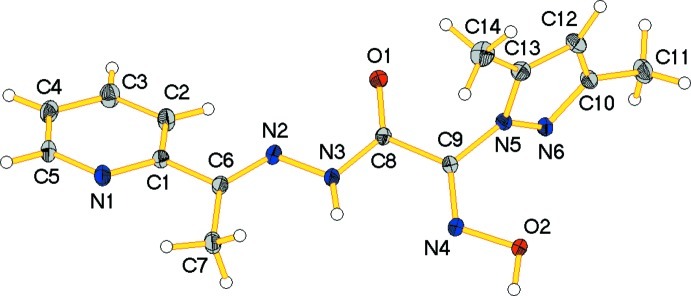
The molecular structure of the title compound, with displacement ellipsoids drawn at the 50% probability level. H atoms are drawn as spheres of arbitrary radii.

**Fig. 2.**
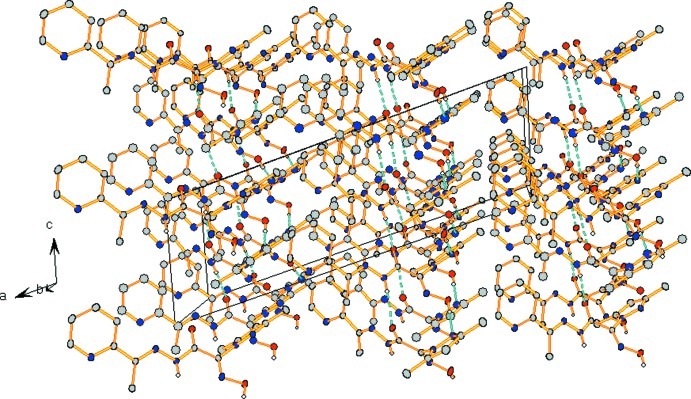
Crystal packing of the title compound. Hydrogen bonds are indicated by dashed lines. H atoms not involved in H-bonds are omitted for clarity.

## supplementary crystallographic information

### Comment

Polynuclear complexes and supramolecular assemblies based on the bridging ligands are widely used in molecular magnetism, crystal engineering, bioinorganic modeling and catalysis (Świątek-Kozłowska *et al.*, 2000; Wörl *et al.*, 2005). One of the most efficient bridging ligands are oximes. Polydentate ligands containing both oxime and other donor functions (*e.g.*, carboxylic, amide, hydroxamic) are of special interest due to their potential for the bridging mode of coordination and mediation of strong magnetic exchange interactions between metal ions (Sachse *et al.*, 2008) and for the preparation of metal complexes with efficient stabilization of unusually high oxidation states of 3d-metal ions (*e.g.*, copper(III) and nickel(III)) (Kanderal *et al.*, 2005). As a part of our research study we present the structure of the title compound, which comprises several donor groups: oxime, hydrazone, azomethine, and pyridine. It has been shown previously that structurally similiar strand ligands form mono- and tetranuclear grid-like assemblies with 3d-metal ions (Moroz *et al.*, 2012).

In the title compound (Fig. 1), the N—N, N—C and C—O bond lengths of the acetohydrazide group (1.3814 (14), 1.3607 (14) and 1.2204 (14) Å respectively) are typical for the protonated amide group (Kanderal *et al.*, 2005; Sachse *et al.*, 2008; Moroz *et al.*, 2012). The NC(=NOH)C(O)NH fragment deviates from the planarity because of a twist between the oxime and the amide groups about the C(8)—C9 bond; the O(1)—C(8)—C(9)—N(4) torsion angle is 168.65 (11)°. The N—O and C—N bond lengths of the oxime group are 1.370 (1) and 1.281 (0) Å, respectively, that is typical for the amide derivatives of 2-hydroxypropanoic acid (Sliva *et al.*, 1997; Mokhir *et al.*, 2002). The pyridine nitrogen atom is situated in an *anti*-position with respect to the azomethine group. The C—C, C—N and N—N' (1.3314 (15)–1.4098 (12) Å) bond lengths in the pyrazole ring have typical values. The N4—C9—N5—N6 torsion angle is 63.95 (15)°. The C—N and C—C bond lengths in the pyridine ring are normal for 2-substituted pyridine derivatives (Krämer & Fritsky, 2000; Sachse *et al.*, 2008).

In the crystal packing (Fig. 2), the molecules are connected by N—H···O and O—H···N hydrogen bonds (Table 1) to form chains parallel to the *c* axis, where the amide nitrogen and the oxime oxygen atoms act as donors and the amide oxygen and the pyrazole nitrogen atoms act as acceptors.

### Experimental

Synthesis of ethyl 2-[1-(3,5-dimethyl)pyrazolyl]-2-hydroxyiminoacetate: a mixture of ethyl 2-chloro-2-hydroxyiminoacetate synthesized according to Kozikowski *et al.* (1983) (0.906 g, 6 mmol) and 3,5-dimethylpyrazole (1.152 g, 12 mmol) in 10 ml of chloroform was left for evaporation in the air overnight. The resulting precipitate was recrystallized from water. Yield: 1.12 g (88%).

Synthesis of 2-[1-(3,5-dimethyl)pyrazolyl]-2-hydroxyiminoacetohydrazide: a solution of hydrazine hydrate (0.57 ml, 60%, 10.6 mmol) in water was added to a solution of ethyl 2-[1-(3,5-dimethyl)pyrazolyl]-2-hydroxyiminoacetate (1.12 g, 5.3 mmol) in methanol (30 ml). The resulting mixture was heating under reflux for 1.5 h. After that the solvent was evaporated and the product was recrystallized from methanol. Yield 0.5 g (48%).

Synthesis of 2-[1-(3,5-dimethyl)pyrazolyl]-2-hydroxyimino-*N*'-[1-(2-pyridyl)ethylidene]acetohydrazide (1): a solution of 2-[1-(3,5-dimethyl)pyrazolyl]-2-hydroxyiminoacetohydrazide (0.5 g, 2.54 mmol) in methanol (30 ml) was treated with 2-acetylpyridine (0.307 g, 2.54 mmol) and the mixture was heated under reflux for 3 h. After that the solvent was evaporated in vacuum and the product was recrystallized from methanol. Yield 0.65 g (85%).

### Refinement

The crystal was refined as a racemic twin, with the BASF value refined to 0.4 (8) for 1715 Friedel pairs. The oxime H atom was located in a difference Fourier map and refined as riding with *U*_iso_ = 1.5 *U*_eq_(O). The hydrazide H atom was also located in a difference Fourier map but not refined; the N—H distance was constrained to be 0.86 (1) Å and the isotropic displacement parameter was set to 1.5 times that of the N parent atom. All other hydrogen atoms were positioned geometrically and constrained to ride on their parent atoms, with C—H = 0.95–0.98 Å, and *U*_iso_ = 1.2–1.5 *U*_eq_(C).

### Crystal data

**Table:**

C_14_H_16_N_6_O_2_	*F*(000) = 632
*M**_r_* = 300.33	*D*_x_ = 1.350 Mg m^−^^3^
Monoclinic, *C**c*	Mo *K*α radiation, λ = 0.71073 Å
Hall symbol: C -2yc	Cell parameters from 5439 reflections
*a* = 24.5792 (6) Å	θ = 3.5–32.2°
*b* = 7.5795 (2) Å	µ = 0.10 mm^−^^1^
*c* = 8.3072 (2) Å	*T* = 100 K
β = 107.335 (1)°	Block, colourless
*V* = 1477.32 (6) Å^3^	0.36 × 0.28 × 0.21 mm
*Z* = 4	

### Data collection

**Table:**

Bruker Kappa APEXII DUO CCD diffractometer	4395 independent reflections
Radiation source: fine-focus sealed tube	4096 reflections with *I* > 2σ(*I*)
Curved graphite crystal monochromator	*R*_int_ = 0.016
Detector resolution: 16 pixels mm^-1^	θ_max_ = 32.2°, θ_min_ = 1.7°
φ scans and ω scans with κ offset	*h* = −28→36
Absorption correction: multi-scan (*SADABS*; Sheldrick, 2008)	*k* = −11→11
*T*_min_ = 0.966, *T*_max_ = 0.980	*l* = −12→12
8846 measured reflections	

### Refinement

**Table:**

Refinement on *F*^2^	Primary atom site location: structure-invariant direct methods
Least-squares matrix: full	Secondary atom site location: difference Fourier map
*R*[*F*^2^ > 2σ(*F*^2^)] = 0.032	Hydrogen site location: inferred from neighbouring sites
*wR*(*F*^2^) = 0.081	H-atom parameters constrained
*S* = 1.03	*w* = 1/[σ^2^(*F*_o_^2^) + (0.0459*P*)^2^ + 0.467*P*] where *P* = (*F*_o_^2^ + 2*F*_c_^2^)/3
4395 reflections	(Δ/σ)_max_ = 0.001
204 parameters	Δρ_max_ = 0.36 e Å^−^^3^
3 restraints	Δρ_min_ = −0.24 e Å^−^^3^

### Special details

**Table:**

Geometry. All e.s.d.'s (except the e.s.d. in the dihedral angle between two l.s. planes) are estimated using the full covariance matrix. The cell e.s.d.'s are taken into account individually in the estimation of e.s.d.'s in distances, angles and torsion angles; correlations between e.s.d.'s in cell parameters are only used when they are defined by crystal symmetry. An approximate (isotropic) treatment of cell e.s.d.'s is used for estimating e.s.d.'s involving l.s. planes.
Refinement. Refinement of *F*^2^ against ALL reflections. The weighted *R*-factor *wR* and goodness of fit *S* are based on *F*^2^, conventional *R*-factors *R* are based on *F*, with *F* set to zero for negative *F*^2^. The threshold expression of *F*^2^ > σ(*F*^2^) is used only for calculating *R*-factors(gt) *etc*. and is not relevant to the choice of reflections for refinement. *R*-factors based on *F*^2^ are statistically about twice as large as those based on *F*, and *R*- factors based on ALL data will be even larger.

### Fractional atomic coordinates and isotropic or equivalent isotropic displacement parameters (Å^2^)

**Table:**

	*x*	*y*	*z*	*U*_iso_*/*U*_eq_	
O1	0.36481 (4)	0.69023 (11)	0.54259 (10)	0.01537 (16)	
O2	0.22703 (4)	0.54704 (12)	0.05549 (11)	0.01825 (18)	
H2O	0.2225	0.4597	−0.0165	0.027*	
N1	0.56071 (4)	0.19215 (14)	0.65828 (13)	0.01662 (19)	
N2	0.42935 (4)	0.41283 (13)	0.53143 (12)	0.01403 (18)	
N3	0.38561 (4)	0.46283 (14)	0.39135 (11)	0.01331 (17)	
H3N	0.3778	0.4153	0.2937	0.019 (4)*	
N4	0.27952 (4)	0.51059 (13)	0.16581 (12)	0.01522 (18)	
N5	0.26314 (4)	0.76793 (12)	0.30874 (11)	0.01181 (17)	
N6	0.21438 (4)	0.73146 (13)	0.35109 (11)	0.01289 (18)	
C1	0.51137 (5)	0.25530 (15)	0.67204 (14)	0.01364 (19)	
C2	0.50044 (5)	0.27091 (17)	0.82763 (15)	0.0172 (2)	
H2	0.4648	0.3142	0.8333	0.021*	
C3	0.54243 (6)	0.22232 (18)	0.97295 (16)	0.0202 (2)	
H3	0.5360	0.2318	1.0799	0.024*	
C4	0.59418 (5)	0.15935 (17)	0.96004 (15)	0.0185 (2)	
H4	0.6240	0.1262	1.0575	0.022*	
C5	0.60086 (5)	0.14650 (17)	0.80058 (15)	0.0179 (2)	
H5	0.6360	0.1026	0.7916	0.021*	
C6	0.46826 (5)	0.30868 (16)	0.51196 (14)	0.01365 (19)	
C7	0.47343 (6)	0.24075 (18)	0.34765 (15)	0.0189 (2)	
H7A	0.4467	0.1428	0.3084	0.028*	
H7B	0.5124	0.1994	0.3634	0.028*	
H7C	0.4645	0.3357	0.2638	0.028*	
C8	0.35246 (5)	0.59735 (15)	0.41689 (13)	0.01189 (19)	
C9	0.29671 (5)	0.62508 (16)	0.28350 (13)	0.01239 (19)	
C10	0.19299 (5)	0.88846 (16)	0.37111 (14)	0.0156 (2)	
C11	0.13847 (6)	0.89937 (19)	0.41554 (17)	0.0226 (3)	
H11A	0.1083	0.9459	0.3192	0.034*	
H11B	0.1435	0.9780	0.5126	0.034*	
H11C	0.1278	0.7815	0.4440	0.034*	
C12	0.22777 (6)	1.02525 (16)	0.34214 (16)	0.0191 (2)	
H12	0.2216	1.1485	0.3478	0.023*	
C13	0.27257 (5)	0.94425 (15)	0.30399 (15)	0.0156 (2)	
C14	0.32359 (6)	1.01682 (18)	0.26595 (17)	0.0227 (3)	
H0AA	0.3555	1.0228	0.3700	0.034*	
H0AB	0.3151	1.1355	0.2182	0.034*	
H0AC	0.3338	0.9401	0.1846	0.034*	

### Atomic displacement parameters (Å^2^)

**Table:**

	*U*^11^	*U*^22^	*U*^33^	*U*^12^	*U*^13^	*U*^23^
O1	0.0150 (4)	0.0166 (4)	0.0135 (4)	0.0014 (3)	0.0026 (3)	−0.0019 (3)
O2	0.0122 (4)	0.0191 (4)	0.0188 (4)	0.0038 (3)	−0.0025 (3)	−0.0061 (3)
N1	0.0132 (4)	0.0191 (5)	0.0162 (4)	0.0036 (4)	0.0025 (4)	0.0002 (4)
N2	0.0111 (4)	0.0165 (4)	0.0129 (4)	0.0010 (3)	0.0012 (3)	0.0014 (3)
N3	0.0116 (4)	0.0160 (4)	0.0110 (4)	0.0033 (3)	0.0013 (3)	−0.0007 (3)
N4	0.0110 (4)	0.0163 (4)	0.0161 (4)	0.0018 (4)	0.0007 (3)	−0.0024 (4)
N5	0.0111 (4)	0.0104 (4)	0.0139 (4)	0.0001 (3)	0.0036 (3)	−0.0001 (3)
N6	0.0108 (4)	0.0134 (4)	0.0147 (4)	0.0011 (3)	0.0040 (3)	0.0013 (3)
C1	0.0126 (5)	0.0130 (5)	0.0145 (5)	0.0012 (4)	0.0028 (4)	0.0005 (4)
C2	0.0148 (5)	0.0221 (6)	0.0151 (5)	0.0044 (4)	0.0052 (4)	0.0023 (4)
C3	0.0199 (6)	0.0250 (6)	0.0153 (5)	0.0036 (5)	0.0048 (5)	0.0035 (4)
C4	0.0166 (5)	0.0189 (5)	0.0168 (5)	0.0021 (4)	0.0001 (4)	0.0041 (4)
C5	0.0119 (5)	0.0203 (5)	0.0197 (5)	0.0045 (4)	0.0018 (4)	0.0005 (4)
C6	0.0113 (5)	0.0155 (5)	0.0133 (5)	0.0008 (4)	0.0024 (4)	0.0002 (4)
C7	0.0161 (5)	0.0242 (6)	0.0151 (5)	0.0072 (5)	0.0027 (4)	−0.0004 (4)
C8	0.0101 (5)	0.0129 (5)	0.0128 (4)	0.0001 (4)	0.0034 (4)	0.0013 (4)
C9	0.0111 (4)	0.0125 (5)	0.0135 (5)	0.0016 (4)	0.0035 (4)	0.0003 (4)
C10	0.0155 (5)	0.0154 (5)	0.0156 (5)	0.0037 (4)	0.0039 (4)	−0.0009 (4)
C11	0.0183 (6)	0.0264 (6)	0.0249 (6)	0.0057 (5)	0.0094 (5)	−0.0016 (5)
C12	0.0215 (6)	0.0115 (5)	0.0237 (6)	0.0026 (4)	0.0056 (5)	−0.0001 (4)
C13	0.0165 (5)	0.0126 (5)	0.0170 (5)	−0.0014 (4)	0.0039 (4)	0.0018 (4)
C14	0.0208 (6)	0.0207 (6)	0.0277 (6)	−0.0052 (5)	0.0087 (5)	0.0048 (5)

### Geometric parameters (Å, º)

**Table:**

O1—C8	1.2204 (14)	C4—C5	1.3859 (17)
O2—N4	1.3697 (13)	C4—H4	0.9500
O2—H2O	0.8763	C5—H5	0.9500
N1—C1	1.3400 (15)	C6—C7	1.4991 (16)
N1—C5	1.3401 (15)	C7—H7A	0.9800
N2—C6	1.2870 (15)	C7—H7B	0.9800
N2—N3	1.3814 (13)	C7—H7C	0.9800
N3—C8	1.3607 (14)	C8—C9	1.4982 (15)
N3—H3N	0.8555	C10—C12	1.4094 (18)
N4—C9	1.2811 (15)	C10—C11	1.4943 (17)
N5—C13	1.3589 (14)	C11—H11A	0.9800
N5—N6	1.3738 (13)	C11—H11B	0.9800
N5—C9	1.4144 (14)	C11—H11C	0.9800
N6—C10	1.3314 (15)	C12—C13	1.3775 (18)
C1—C2	1.4013 (16)	C12—H12	0.9500
C1—C6	1.4884 (15)	C13—C14	1.4870 (18)
C2—C3	1.3841 (17)	C14—H0AA	0.9800
C2—H2	0.9500	C14—H0AB	0.9800
C3—C4	1.3922 (18)	C14—H0AC	0.9800
C3—H3	0.9500		
			
N4—O2—H2O	102.0	H7A—C7—H7B	109.5
C1—N1—C5	117.66 (10)	C6—C7—H7C	109.5
C6—N2—N3	118.91 (9)	H7A—C7—H7C	109.5
C8—N3—N2	115.22 (9)	H7B—C7—H7C	109.5
C8—N3—H3N	119.2	O1—C8—N3	123.90 (10)
N2—N3—H3N	125.6	O1—C8—C9	119.44 (10)
C9—N4—O2	113.87 (9)	N3—C8—C9	116.61 (9)
C13—N5—N6	112.00 (9)	N4—C9—N5	123.87 (10)
C13—N5—C9	129.53 (10)	N4—C9—C8	119.37 (10)
N6—N5—C9	118.41 (9)	N5—C9—C8	116.30 (9)
C10—N6—N5	105.03 (9)	N6—C10—C12	110.72 (10)
N1—C1—C2	122.41 (11)	N6—C10—C11	119.80 (11)
N1—C1—C6	116.25 (10)	C12—C10—C11	129.47 (11)
C2—C1—C6	121.34 (10)	C10—C11—H11A	109.5
C3—C2—C1	118.94 (11)	C10—C11—H11B	109.5
C3—C2—H2	120.5	H11A—C11—H11B	109.5
C1—C2—H2	120.5	C10—C11—H11C	109.5
C2—C3—C4	119.01 (11)	H11A—C11—H11C	109.5
C2—C3—H3	120.5	H11B—C11—H11C	109.5
C4—C3—H3	120.5	C13—C12—C10	106.18 (10)
C5—C4—C3	117.96 (11)	C13—C12—H12	126.9
C5—C4—H4	121.0	C10—C12—H12	126.9
C3—C4—H4	121.0	N5—C13—C12	106.06 (10)
N1—C5—C4	124.00 (11)	N5—C13—C14	122.13 (11)
N1—C5—H5	118.0	C12—C13—C14	131.80 (11)
C4—C5—H5	118.0	C13—C14—H0AA	109.5
N2—C6—C1	114.35 (9)	C13—C14—H0AB	109.5
N2—C6—C7	126.36 (10)	H0AA—C14—H0AB	109.5
C1—C6—C7	119.29 (10)	C13—C14—H0AC	109.5
C6—C7—H7A	109.5	H0AA—C14—H0AC	109.5
C6—C7—H7B	109.5	H0AB—C14—H0AC	109.5

### Hydrogen-bond geometry (Å, º)

**Table:**

*D*—H···*A*	*D*—H	H···*A*	*D*···*A*	*D*—H···*A*
O2—H2*O*···N6^i^	0.88	1.79	2.6686 (13)	175
N3—H3*N*···O1^i^	0.86	2.17	3.0196 (13)	174

Symmetry code: (i) *x*, −*y*+1, *z*−1/2.
